# The acidic tumor microenvironment enhances PD-L1 expression via activation of STAT3 in MDA-MB-231 breast cancer cells

**DOI:** 10.1186/s12885-022-09956-9

**Published:** 2022-08-04

**Authors:** Yong-Jin Kwon, Eun-Bi Seo, Ae Jin Jeong, Song-Hee Lee, Kum Hee Noh, Sangsik Lee, Chung-Hyun Cho, Chang-Han Lee, Hyun Mu Shin, Hang-Rae Kim, Hyeong-Gon Moon, Sang-Kyu Ye

**Affiliations:** 1 Department of Pharmacology and Biomedical Sciences, Seoul National University College of Medicine, Seoul, 03080 Republic of Korea; 2 Biomedical Science Project (BK21PLUS), Seoul National University College of Medicine, Seoul, 03080 Republic of Korea; 3 Department of Biomedical Engineering, Catholic Kwangdong University College of Medical Convergence, Gangneung, 25601 Republic of Korea; 4 Wide River Institute of Immunology, Seoul National University, Hongcheon, 25159 Republic of Korea; 5 Department of Anatomy and Cell Biology, Seoul National University College of Medicine, Seoul, 03080 Republic of Korea; 6 Department of Surgery, Seoul National University College of Medicine, Seoul, 03080 Republic of Korea; 7 Ischemic/Hypoxic Disease Institute, Seoul National University College of Medicine, Seoul, 03080 Republic of Korea; 8 Neuro-Immune Information Storage Network Research Center, Seoul National University College of Medicine, Seoul, 03080 Republic of Korea

**Keywords:** Extracellular acidosis, Immune checkpoint, PD-L1, STAT3, MDA-MB-231 cells

## Abstract

Tumor acidosis, a common phenomenon in solid cancers such as breast cancer, is caused by the abnormal metabolism of cancer cells. The low pH affects cells surrounding the cancer, and tumor acidosis has been shown to inhibit the activity of immune cells. Despite many previous studies, the immune surveillance mechanisms are not fully understood. We found that the expression of PD-L1 was significantly increased under conditions of extracellular acidosis in MDA-MB-231 cells. We also confirmed that the increased expression of PD-L1 mediated by extracellular acidosis was decreased when the pH was raised to the normal range. Gene set enrichment analysis (GSEA) of public breast cancer patient databases showed that PD-L1 expression was also highly correlated with IL-6/JAK/STAT3 signaling. Surprisingly, the expression of both phospho-tyrosine STAT3 and PD-L1 was significantly increased under conditions of extracellular acidosis, and inhibition of STAT3 did not increase the expression of PD-L1 even under acidic conditions in MDA-MB-231 cells. Based on these results, we suggest that the expression of PD-L1 is increased by tumor acidosis via activation of STAT3 in MDA-MB-231 cells.

**Supplementary Information:**

The online version contains supplementary material available at 10.1186/s12885-022-09956-9.

## Introduction

Extracellular acidosis is commonly observed in solid cancers. Pyruvate is converted to lactic acid by lactate dehydrogenase (LDH) under anaerobic conditions; in cancer cells, the Warburg effect, in which pyruvic acid is converted to lactic acid under aerobic conditions, also occurs [1, 2]. Excessive production of lactic acid results in its extracellular release, which in turn results in extracellular acidosis [3]. The low pH of the area surrounding the tumor is known to promote metastasis and proliferation by inducing an aggressive phenotype, as well as by suppressing immune cell activity [4]. Therefore, targeting extracellular acidosis to suppress cancer cell activity and activate immune cells is essential for cancer treatment.

The immune checkpoint proteins programmed cell death protein-1 (PD-1) and programmed cell death ligand-1 (PD-L1) are mainly expressed on immune cells and cancer cells, respectively [5]. When bound by PD-L1 expressed on cancer cells, PD-1 of immune cells is exhausted and cannot effectively target cancer cells [6]. PD-L1 expression is increased in cancer cells; higher PD-L1 expression in cancer patients is correlated with significantly lower overall and recurrence-free survival. Thus, targeting the increased expression of PD-L1 is important for immunotherapy [7, 8].

When phosphorylated, the transcription factor signal transducer and activator of transcription 3 (STAT3) forms homo or heterodimers, and translocates from the cytoplasm to the nucleus to induce the expression of target genes [9]. The increased activation of STAT3 in cancer cells results in the expression of various oncogenes that induce tumor metastasis, stemness, proliferation, angiogenesis, and anti-apoptosis [10]. In addition, STAT3 induces the expression of PD-L1, which has been linked to immunosuppression [11]. Therefore, activating STAT3 by suppressing immune exhaustion through inhibition of PD-L1 expression may be a viable strategy for cancer treatment.

Recent studies have reported that the expression of PD-L1 is increased by lactate in Lewis lung carcinoma and melanoma cells; tumor growth was also significantly reduced following treatment with both sodium bicarbonate (NaHCO_3_) and a PD-L1 inhibitor [12–14]. However, the relationship between tumor acidosis and PD-L1 in breast cancer, and the detailed mechanisms by which PD-L1 is increased by acidosis, are not well understood. The aim of this study was to identify the cause of the increased PD-L1 expression, and to suggest novel methods to suppress PD-L1 expression in breast cancer cells.

Although many drugs have been developed to inhibit PD-L1, it is important to understand the underlying cause of increased PD-L1 expression in cancer cells [15–17]. We found that extracellular acidosis increased PD-L1 expression in MDA-MB-231 cells. In addition, tyrosine 705 of STAT3 was significantly phosphorylated by acidification, and the expression of PD-L1 was increased by phosphorylation of STAT3. The results of this study suggest that targeting tumor acidosis could prevent immune exhaustion in breast cancer cells.

## Materials and methods

### Reagents and antibodies

Sodium bicarbonate (NaHCO_3_), lactic acid, sodium lactate and sodium oxamate were obtained from Sigma Aldrich (St. Louis, MO, USA). The antibodies used in this study were as follows: anti-PD-L1 (Abcam, Cambridge, UK); anti-LDH-A (Santa Cruz Biotechnology, Santa Cruz, CA, USA); anti-pY^701^-STAT1, anti-STAT1, anti-pY^705^-STAT3, anti-pS^727^-STAT3, anti-STAT3 and anti-β-actin (Cell Signaling Technology, Danvers, MA, USA); and anti-HIF-1α (Abbkine, Wuhan, China).

### Cell culture and culture conditions

The human breast cancer cell line MDA-MB-231 was obtained from the American Type Culture Collection (Manassas, VA, USA), and Dulbecco’s modified Eagle’s medium (DMEM), fetal bovine serum (FBS), and penicillin/streptomycin were obtained from Capricorn Scientific (Ebsdorfergrund, Germany). MDA-MB-231 cells were maintained in DMEM with 10% FBS and 1% penicillin/streptomycin and incubated in a humidified incubator (Vision Science, Seoul, Republic of Korea) containing 5% CO_2_ at 37 °C.

### pH regulation and measure

To mimic extracellular acidosis, 1 M HCl and lactic acid were used as described previously [18]. In addition, 1 M NaOH and NaHCO_3_ were used to neutralize or buffer acidic pH. The pH-adjusted culture medium was stabilized in a 5% CO_2_ incubator prior to treatment. The pH of cell culture media was measured using a pH meter (Mettler Toledo, Columbus, OH, USA).

### Conditioned medium

MDA-MB-231 cells were cultured in normoxia (21% O_2_ and 5% CO_2_) or hypoxia (2% O_2_ and 5% CO_2_) with/without oxamate for 36 h, and normoxic conditioned medium (NCM) and hypoxic conditioned medium (HCM) were collected. Heat-inactivated HCM was obtained by boiling HCM for 20 min at 100 °C to degrade secretory proteins in the conditioned medium, and the pH neutralized HCM was obtatined by adding 1 M NaOH to neutralize acidified HCM.

### Western blotting

MDA-MB-231 cells were lysed using 1% Triton X-100 lysis buffer containing protease and phosphatase inhibitors (2 mM PMSF, 1 mM NaF, 2 mM EDTA, 0.5 mM Na_3_VO_4_ and 10 μg/mL leupeptin). Cell lysates were separated by sodium dodecyl sulfate-polyacrylamide gel electrophoresis and transferred onto a nitrocellulose membrane (GE Healthcare Life Sciences, Chicago, IL, USA). The membranes were blocked with 5% skim-milk (LPS Solution, Daejeon, Repulic of Korea) for 1 h. The blocked membranes were incubated with primary antibodies (1:1000–2000) at 4 °C overnight followed by horseradish peroxidase-conjugated secondary antibodies (1:5000–10,000) at room temperature for 2 h. The membranes were incubated with ECL reagents (BioMax, Seoul, Republic of Korea) and developed on AGFA CP-BU New X-ray film (Agfa-Gevaert N.V., Mortsel, Belgium). Densitometric and statistical analysis of western blot bands are included in the Supplementary Fig. 5 and 6, and all western blot band images are included in the Supplementary Fig. 7–13.

### Quantitative real-time polymerase chain reaction (qPCR) analysis

Total RNA was extracted using the RNAiso Plus (total RNA extraction reagent; Takara, Shiga, Japan) and cDNA was synthesized using the ReverTra Ace qPCR RT Master Mix (TOYOBO, Osaka, Japan) according to the manufacturer’s protocols. To compare the mRNA expression of target genes, qPCR was performed using the BlasTaq qPCR MasterMix (Applied Biological Materials, Richmond, Canada), and the fluorescent signal of individual target genes was detected using a CFX Connect Real-Time PCR Detection System (Bio-Rad Laboratories, Hercules, CA, USA). The qPCR primers used in this study were as follows: PD-L1 forward, 5′- AAATGGAACCTGGCGAAAGC − 3′, and PD-L1 reverse, 5′- GATGAGCCCCTCAGGCATTT -3′; PD-L2 forward, 5′- GTCTTGGGAGCCAGGGTGAC − 3′, and PD-L2 reverse, 5′- TGAAAAGTGCAAATGGCAAGC -3′; GAPDH forward, 5′- CTGACTTCAACAGCGACACC − 3′, and GAPDH reverse, 5′- TAGCCAAATTCGTTGTCATACC − 3′.

### Plasmids and small interfering-RNA (si-RNA) transfection

STAT3 was overexpressed with pcDNA3.1-GFP and pcDNA3.1-wild type STAT3-GFP vectors using Lipofectamine 3000 (Invitrogen, Waltham, MA, USA) for 48 h. STAT3 was knocked down with human si-STAT3 (SI02662338; Qiagen, Hilden, Germany) and a negative control si-RNA (1,027,280; Qiagen) using RNAiMax (Invitrogen) for 48 h. Transfection experiments were performed according to the manufacturer’s protocol (https://www.thermofisher.com).

### Immunohistochemistry (IHC)

Human breast tissues, provided by Seoul National University Hospital with approval from the SNUH Institutional Review Board (IRB; IRB No. 1904–141-1029), were fixed in 4% paraformaldehyde for 24 h at 4 °C and embedded in paraffin. The paraffin blocks were cut into 4-μm-thick sections, and the paraffin slides were deparaffinized with xylene and hydrated with graded ethanol. Antigen was unmasked by boiling the sections in 100 mM citrate buffer (pH 6.0) for 10 min and endogenous peroxidase activity was blocked with 3% hydrogen peroxide for 10 min. The slides were blocked in 5% normal goat serum (Vector Laboratories, Burlingame, CA, USA) for 1 h and incubated with primary antibodies (diluted 1:100–200 in antibody diluent solution; Life Technologies, Carlsbad, CA, USA) overnight at 4 °C. The following day, sections were incubated with biotin-conjugated secondary antibodies (diluted 1:100–200 in antibody diluent solution; Vector Laboratories) for 1 h and incubated with avidin-biotin complex reagent for 1 h. The slides were visualized using DAB staining solution (Dako, Santa Clara, CA, USA) followed by counterstaining with hematoxylin solution (Dako). Images of stained sections were obtained using the LAS microscope (Leica Microsystem, Wetzlar, Germany).

### Public database analysis

The public datasets used in this study were GSE12276, GSE27830, GSE5460, GSE54002, and GSE36295 (https://www.ncbi.nlm.nih.gov/geo/). Gene set enrichment analysis (GSEA) was perform to analyze enrichment plots of tumor hallmark gene sets of breast cancer patients (https://www.gsea-msigdb.org) by dividing the PD-L1 high and low expression groups in the GSE12276, GSE27830 and GSE5460 data sets. *PD-L1* and *LDH-A* mRNA expression in human normal breast and breast cancer tissues were comparatively analyzed using the GSE54002 and GSE36295 data sets.

### Statistical analysis

Statistical analysis of all data was performed using Microsoft Excel 2016 software (Microsoft Corp., Redmond, WA, USA) and GraphPad Prism 5 (GraphPad Software Inc., La Jolla, CA, USA). Results are presented as the mean ± standard deviation of at least three independent experiments. Unpaired Student’s *t*-test was used for analyzing the data, *p*-values of < 0.05, < 0.01, and < 0.001 were considered statistically significant.

## Results

### Extracellular acidosis increases PD-L1 expression in MDA-MB-231 cells

**Fig. 1:**
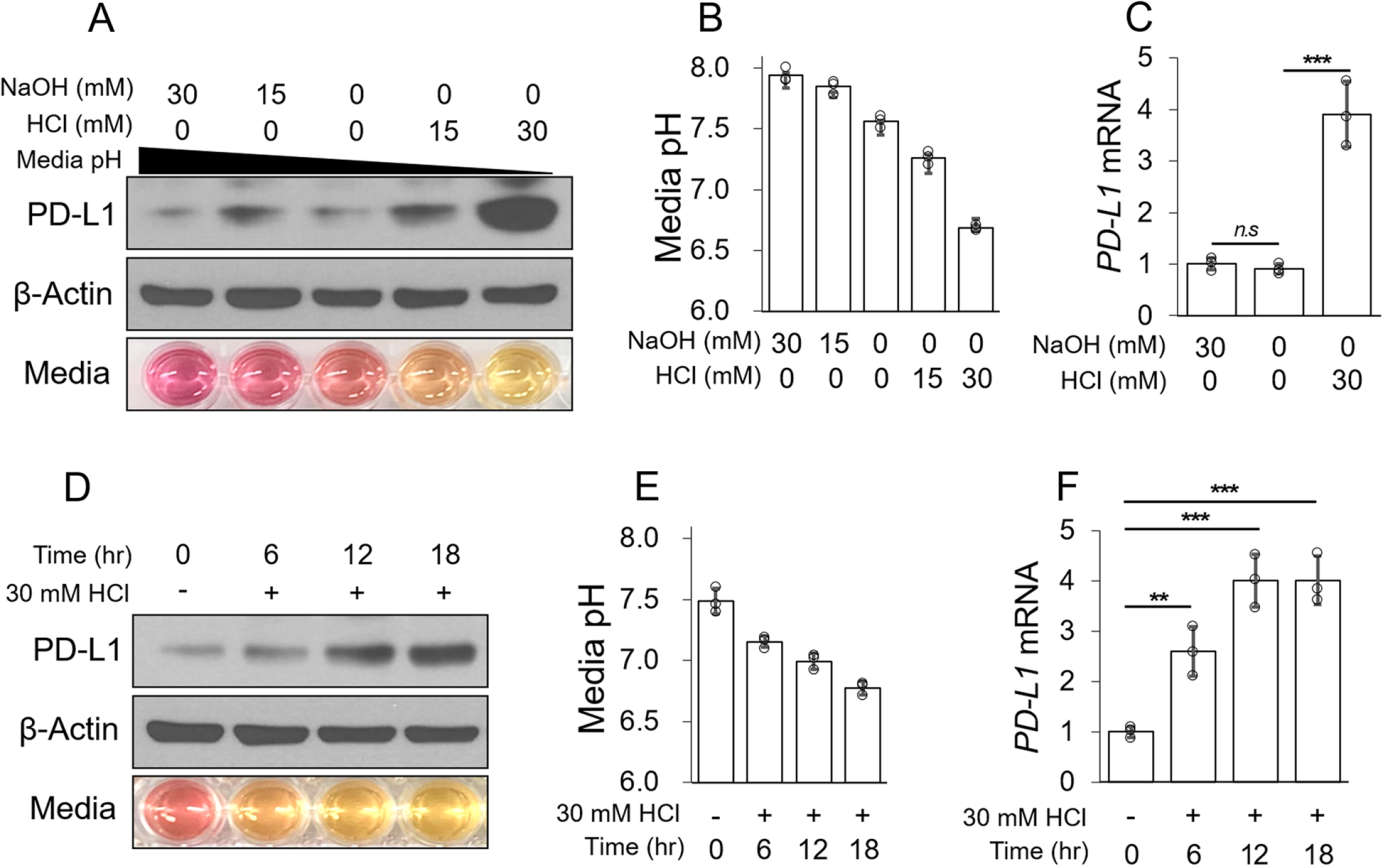
Extracellular acidosis increases PD-L1 expression in MDA-MB-231 cells. **A–C** The pH of the medium was adjusted by HCl or NaOH, and MDA-MB-231 cells were incubated for 18 h. Protein levels were analyzed by western blotting (**A**), the culture medium pH was measured using a pH meter (**B**), and mRNA levels were analyzed by qPCR **(C)**. **D–F** MDA-MB-231 cells were incubated under acidic conditions for the indicated periods. Protein levels were analyzed by western blotting (**D**), culture medium pH was measured using a pH meter (**E**), and mRNA levels were analyzed by qPCR (**F**)

To confirm the relationship between tumor acidosis and PD-L1 expression in breast cancer cells, we adjusted the pH of the medium using HCl or NaOH (Supplementary Fig. 1A). Protein and mRNA levels of PD-L1 were significantly increased at the acidic pH (Fig. 1A–C). When PD-L2 expression by tumor acidosis was confirmed, PD-L1 expression increased approximately 3–4-fold, while PD-L2 increased up to 1.5-fold; PD-L1 expression was higher than that of PD-L2 (Supplementary Fig. 1B). A time course analysis revealed that the protein level of PD-L1 was significantly increased after 12 h by acidic pH, and the mRNA level was significantly increased after 6 h. These results indicate that the protein and mRNA levels of PD-L1 are significantly increased by acidic pH in MDA-MB-231 cells.

### Tumor-derived lactic acidosis increases PD-L1 expression in MDA-MB-231 cells

**Fig. 2:**
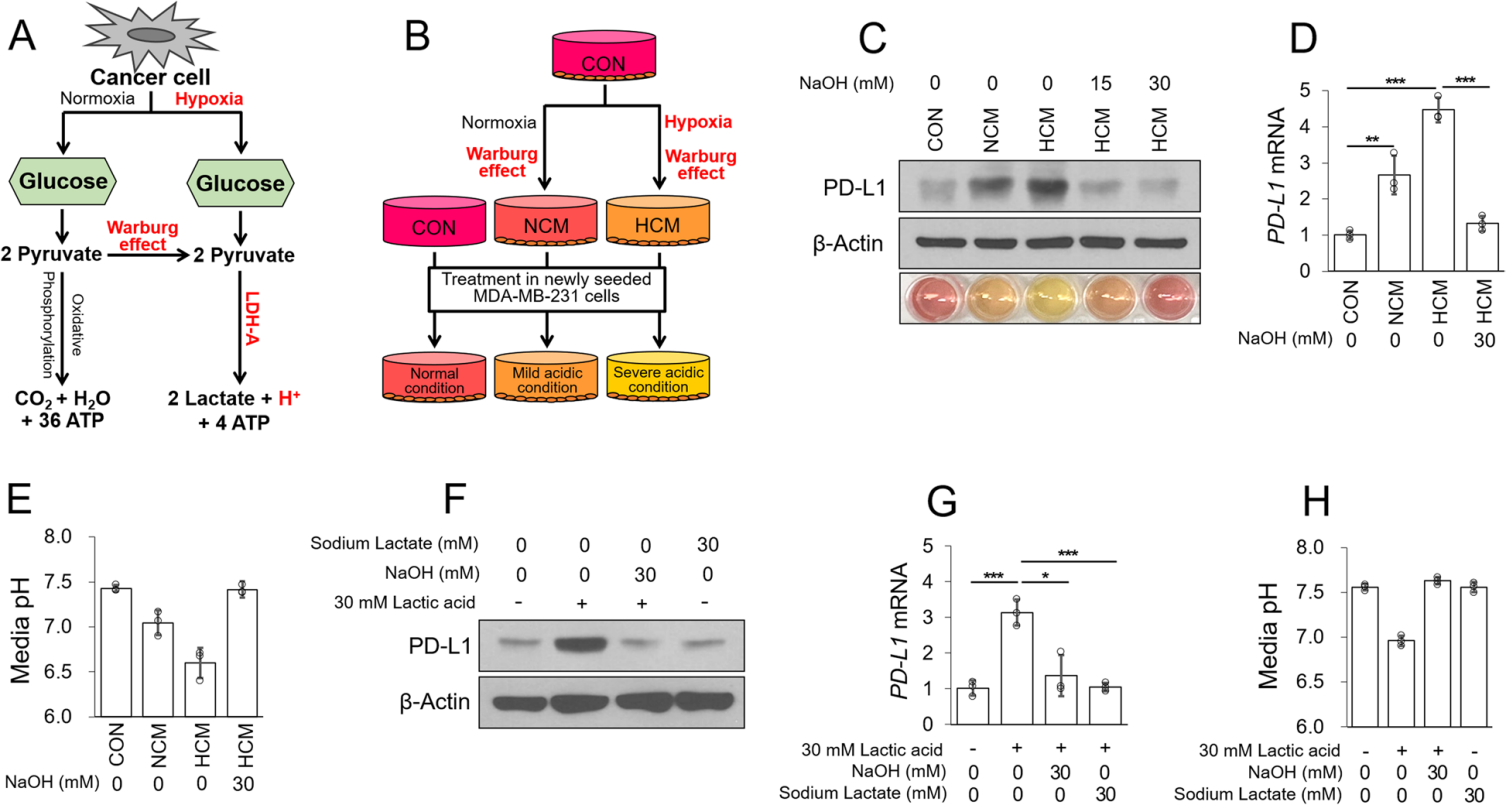
Tumor-derived lactic acidosis increases PD-L1 expression in MDA-MB-231 cells. **A** Schematic diagram of the glycolysis pathway in cancer cells. **B** Summary of the process through which various conditioned media were prepared (CON, control; fresh medium), (NCM, normoxic conditioned medium), (HCM, hypoxic conditioned medium). **C–E** MDA-MB-231 cells were treated with various conditioned media with/without NaOH for 18 h. Protein levels were analyzed by western blotting (**C**), mRNA levels were analyzed by qPCR (**D**), and the culture medium pH was measured using a pH meter (**E**)*.* **F**–**H** MDA-MB-231 cells were treated with lactic acid or sodium lactate, with/without, NaOH for 24 h. Protein levels were analyzed by western blotting (**F**), mRNA levels were analyzed by qPCR (G), and the culture medium pH was measured using a pH meter (**H**)

Extracellular acidosis occurs in a hypoxic environment and/or via the Warburg effect in cancer [1, 2]. In these tumor microenvironments, cancer cells excessively convert pyruvate to lactic acid and ATP by overexpressing LDH-A to produce energy [19]. As a result, a large amount of lactic acid is released from the cells and the pH around them is lowered (Fig. 2A). We predicted that expression of PD-L1 would be increased in cancer cells as a result of lactic acid-induced acidosis. To mimic the lactic acid acidification caused by the Warburg effect and hypoxic environment in cancer, NCM obtained by the Warburg effect under normoxic conditions and HCM obtained by the Warburg effect under hypoxic conditions were prepared. Newly seeded cells were treated with these conditioned media (Fig. 2B). Under both the mildly and severely acidic conditions (NCM and HCM, respectively), protein and mRNA levels of PD-L1 were significantly increased by lactic acidosis, but neutralization of HCM by NaOH did not increase PD-L1 expression (Fig. 2C–E). We also confirmed that PD-L1 expression was increased following treatment with heat-inactivated HCM, suggesting that hypoxia-induced cytokines and/or chemokines did not affect the expression of PD-L1 in MDA-MB-231 cells (Supplementary Fig. 2A–C). As we expected PD-L1 expression to be increased by the Warburg effect and hypoxia-induced lactic acidosis, we directly treated MDA-MB-231 cells with lactic acid. Direct treatment with lactic acid resulted in a decrease in the pH of the medium, and increased protein and mRNA levels of PD-L1. However, sodium lactate, which did not regulate the pH of the medium, did not affect PD-L1 expression (Fig. 2F–H and Supplementary Fig. 2D, E). These results suggest that tumor-derived lactic acidosis is a major cause of the increased expression of PD-L1 in MDA-MB-231 cells.

### Acidosis-induced PD-L1 expression is attenuated by targeting the acidic pH of MDA-MB-231 cells

**Fig. 3:**
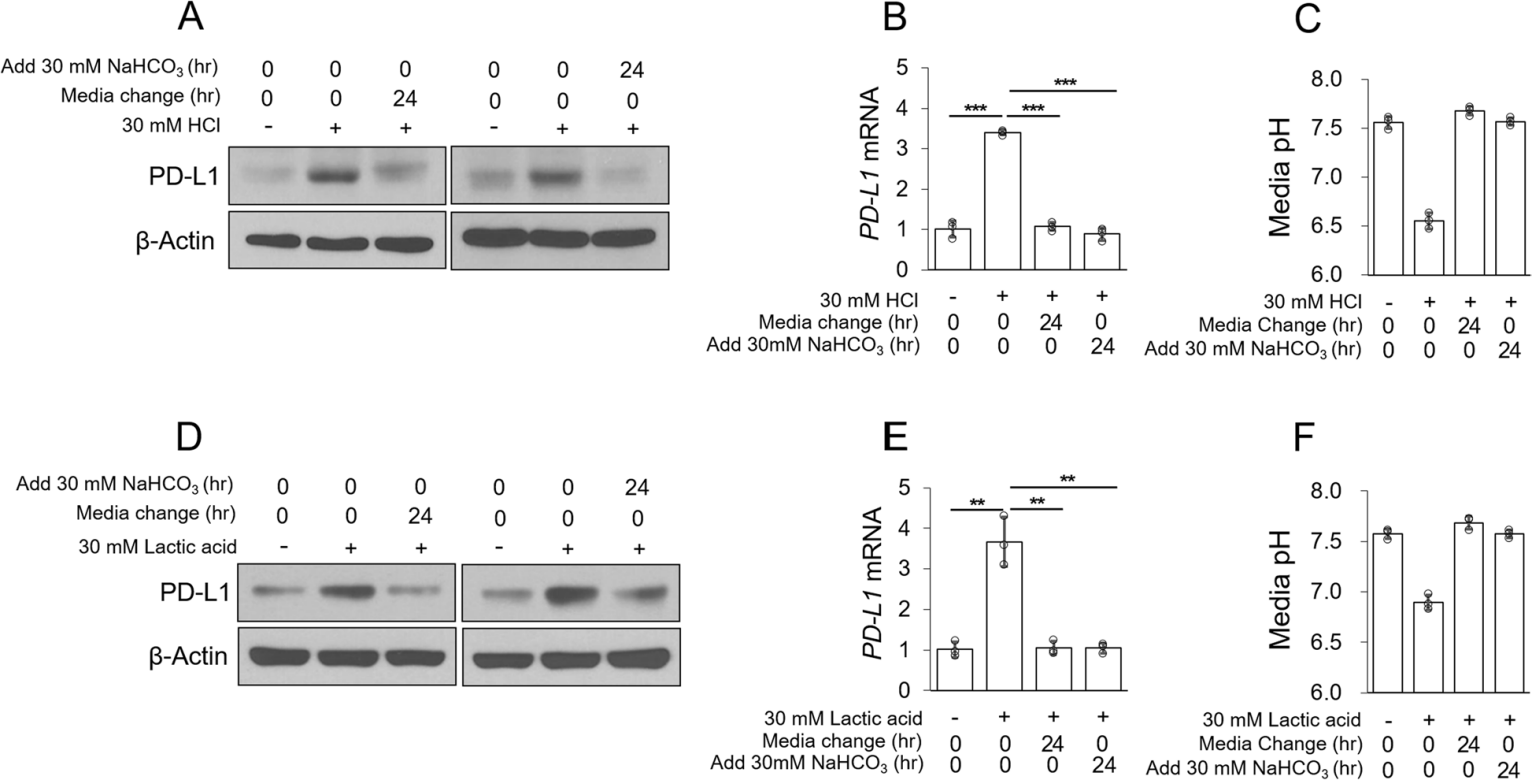
Acidosis-induced PD-L1 expression is attenuated by targeting the acidic pH of MDA-MB-231 cells. **A–C** MDA-MB-231 cells were incubated with HCl-treated acidic medium for 18 h; the acidic culture medium was replaced with fresh medium, or NaHCO_3_ was added for 24 h. Protein levels were analyzed by western blotting **(A)**, mRNA levels were analyzed by qPCR (**B**), and the culture medium pH was measured using a pH meter (**C**). **D–F** MDA-MB-231 cells were incubated with lactic acid-treated acidic medium for 24 h; the acidic culture medium was replaced with fresh medium or NaHCO_3_ was added for 24 h. Protein levels were analyzed by western blotting (**D**), mRNA levels were analyzed by qPCR (**E**), and the culture medium pH was measured using a pH meter (**F**)

When PD-L1 of cancer cells binds PD-1 of immune cells, immune cells become exhausted and cancer cells exhibit immune escape [6]. Therefore, targeting overexpressed PD-L1 in cancer cells may be a viable therapeutic strategy for cancer treatment. Since the expression of PD-L1 is increased by acidic pH, we wondered whether PD-L1 expression would be lowered if the acidic pH was restored to normal levels. The expression of PD-L1 was lowered when the acidic medium was replaced with fresh medium or the acidic medium was buffered with NaHCO_3_ (Fig. 3A–C). The lactic acid-induced increase in PD-L1 expression was also reduced when the acidic medium was replaced with fresh medium or acidic medium buffered with NaHCO_3_ (Fig. 3D–F). Based on these results, we suggest that the expression of acidosis-induced PD-L1 is attenuated when the acidic pH is adjusted to a normal pH in MDA-MB-231 cells.

### Increased PD-L1 expression is correlated with STAT3 activation under acidic conditions in MDA-MB-231 cells

**Fig. 4:**
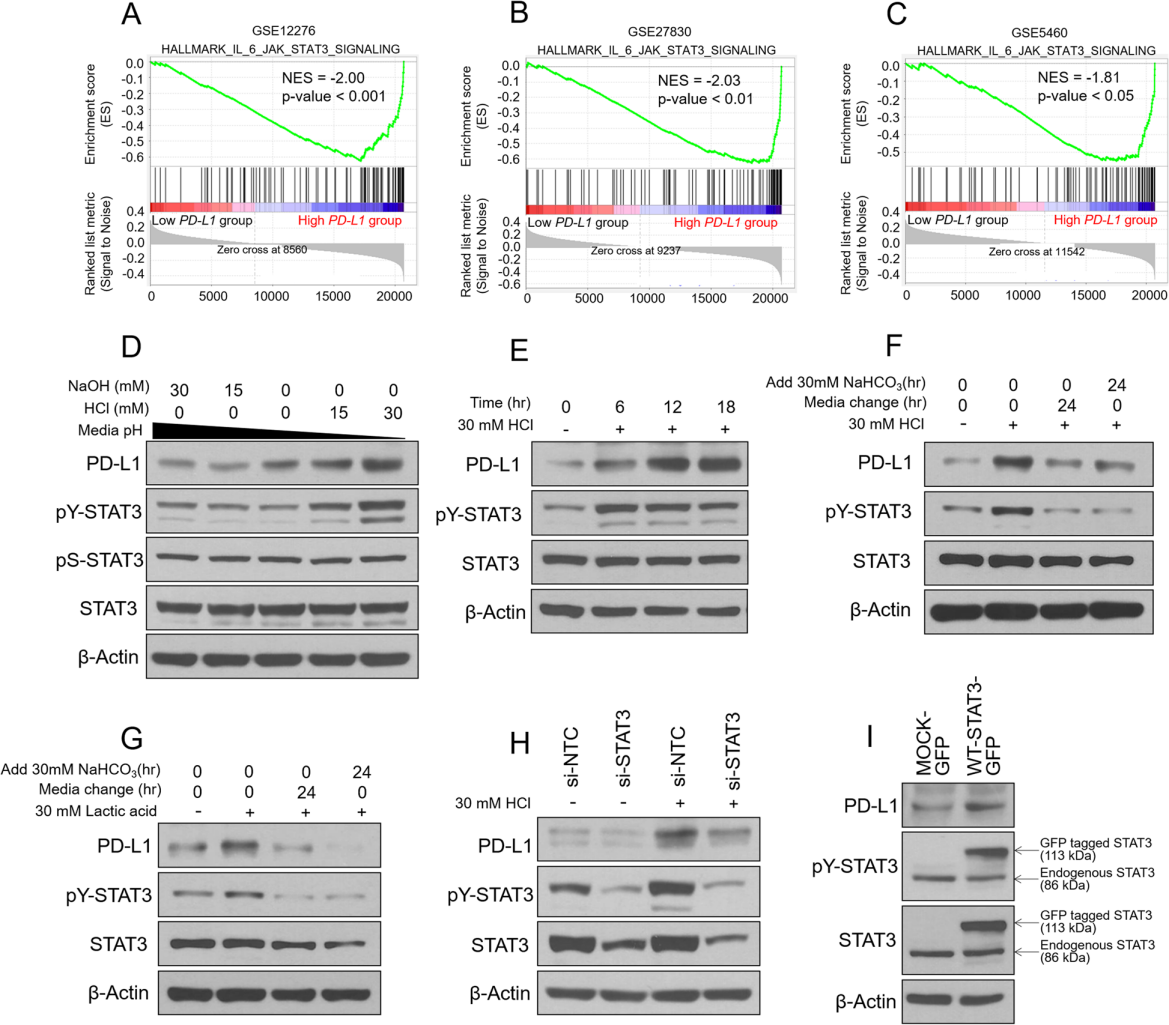
Increased PD-L1 expression is correlated with activation of STAT3 under acidic conditions in MDA-MB-231 cells. **A–C** Gene set enrichment analysis (GSEA) results according to high and low expression of PD-L1 in breast cancer patients. **D** The pH of the medium was adjusted with HCl or NaOH, and MDA-MB-231 cells were treated for 18 h. Protein levels were analyzed by western blotting. **E** MDA-MB-231 cells were incubated under acidic conditions for the indicated periods, and protein levels were analyzed by western blotting. **F, G** MDA-MB-231 cells were incubated with HCl or lactic acid-treated acidic medium for 18 or 24 h; the acidic culture medium was replaced with new medium or NaHCO_3_ was added for 24 h. Protein levels were analyzed by western blotting. **H** MDA-MB-231 cells were transfected with si-NTC (negative control) or si-STAT3 for 24 h followed by treatment with HCl-treated acidic medium for 18 h. Protein levels were analyzed by western blotting. **I** MDA-MB-231 cells were transfected with mock-GFP or WT-STAT3-GFP for 48 h. Protein levels were analyzed by western blotting

We decided to further investigate the tumor acidosis-induced increase in PD-L1 expression. GSEA of public breast cancer patient databases confirmed that IL-6/JAK/STAT3 signaling was significantly correlated with PD-L1 expression (Fig. 4A–C). Previous studies have reported that activation of STAT3 is related to PD-L1 expression in various cancers, including breast cancer [11, 20]. Interestingly, when MDA-MB-231 cells were treated with pH-dependent medium in a time-dependent manner, pS-STAT3 was unchanged while pY-STAT3 was significantly increased under acidic conditions (Fig. 4D, E). NCM, HCM, and lactic acid also increased pY-STAT3 levels in a similar manner as PD-L1, but not when the acidic pH was neutralized with NaOH or sodium lactate (Supplementary Fig. 3A, B). The increased expression of pY-STAT3 induced by acidosis or lactic acidosis was attenuated by replacing or buffering the acidic pH, as with PD-L1 (Fig. 4F, G). In addition, when oxamate, an LDH inhibitor, was treated under hypoxic conditions, hypoxia-induced lactic acidosis was suppressed; oxamate pre-treated HCM did not increase pY-STAT3 and PD-L1 expression (Supplementary Fig. 3C-F). Since PD-L1 is a target gene of STAT3, we expected that inhibition of STAT3 in tumor acidosis would inhibit the expression of PD-L1. As expected, levels of PD-L1 and pY-STAT3 were not increased under acidic conditions when STAT3 was silenced by siRNA, or inhibited with the STAT3 inhibitor nifuroxazide (Fig. 4H and Supplementary Fig. 3G), and PD-L1 expression was increased when STAT3 was overexpressed (Fig. 4I). These results suggest that STAT3 activation by tumor acidosis is a major factor underlying increased PD-L1 expression.

### Clinical relevance of PD-L1 expression in human breast cancer patients

**Fig. 5:**
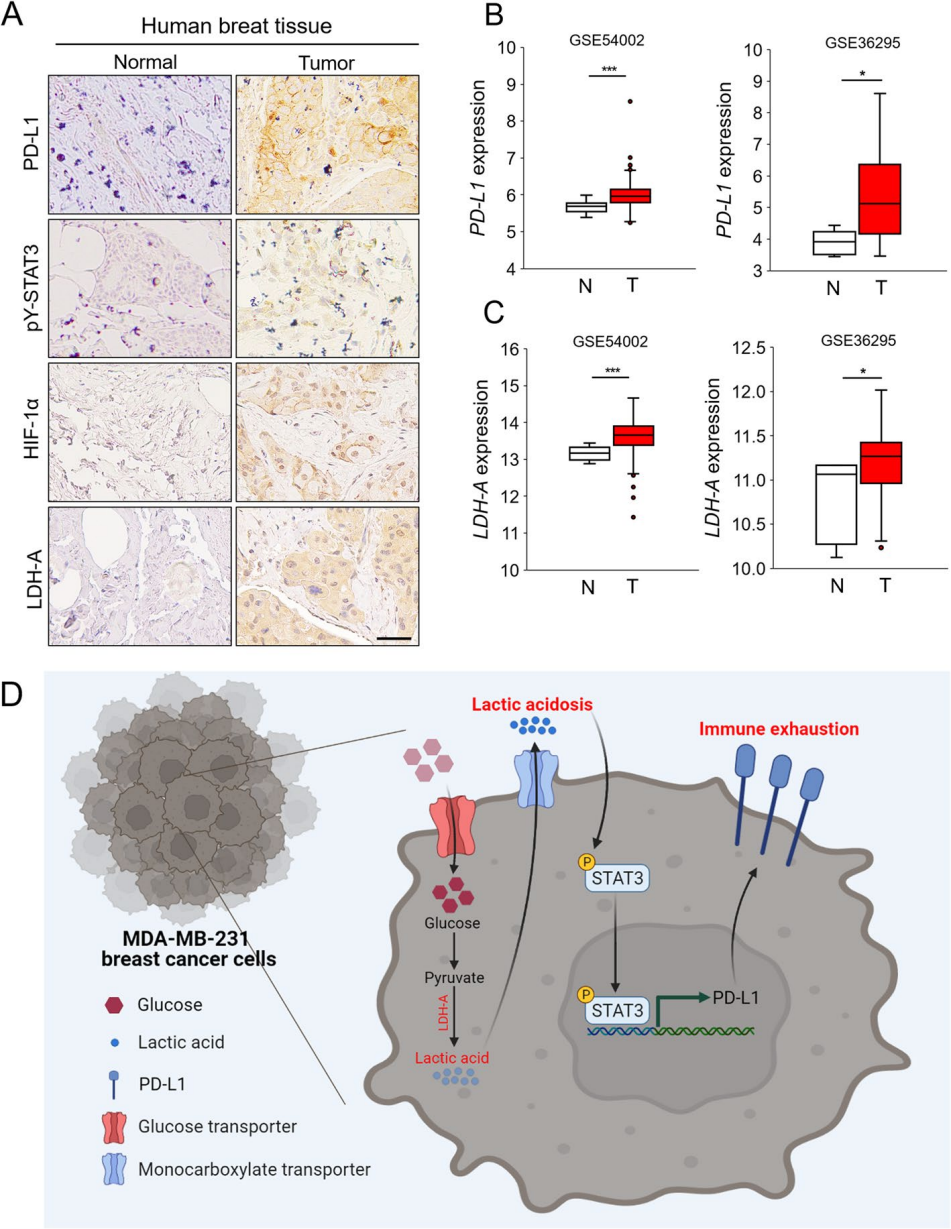
Clinical relevance of PD-L1 expression in human breast cancer patients. **A** Immunohistochemical analysis of normal and tumor breast tissues. Scale bars: 50 μm. **B**, **C** mRNA expression of PD-L1 (**B**) and LDH-A (**C**) in normal and tumor breast tissues from GEO databases. N, normal tissue; T, tumor tissue. **D** Graphical summary adapted from *“Cancer cell”* by BioRender.com*(2021)* and retrieved from https://app.biorender.com/biorender-templates

To demonstrate the clinical relevance of PD-L1 expression and tumor acidosis, we determined the correlations among PD-L1, STAT3, and acidic microenvironment markers by analyzing breast cancer tissue. The expression of LDH-A, an enzyme important in the production of lactic acid, and hypoxia-inducible factor-1α (HIF-1α), a major marker of hypoxia, was increased in breast cancer cells compared with normal breast cells; the expression of PD-L1 and pY-STAT3 was also increased in breast cancer cells (Fig. 5A). Using the GEO databases, we confirmed that the expression of PD-L1 and LDH-A was higher in cancer cells than normal cells (Fig. 5B, C). These results suggest that tumor acidosis is positively correlated with PD-L1 expression.

## Discussion

Hypoxia, a typical tumor microenvironment, is seen in most solid cancers. In the absence of oxygen, glucose is not completely consumed by oxidative phosphorylation through mitochondria, or by LDH, which produces lactic acid as a byproduct [2, 4]. In cancer cells, the expression of monocarboxylate transporters (MCTs) such as MCT1 and MCT4, is increased to allow release of the lactic acid that accumulates in cells. When a large amount of lactic acid is released, the extracellular pH is lowered [21]. Tumor acidosis promotes cancer progression, and various attempts have been made to inhibit this phenomenon, such as inhibiting intracellular LDH-A and lactic acid transporters, and directly administering a pH buffer such as NaHCO_3_ [22–24]. In addition, the development of pH-sensitive drug delivery systems to specifically target anti-cancer agents in cancer cells is under investigation [25]. Neutralizing the acidic pH of cancer cells can inhibit cancer progression, and synergistic effects of neutralization of the acidic pH and treatment with anti-cancer drugs have been reported [14, 26–29]. It is essential to understand the different cancer microenvironments and exploit the effects of cancer acidification.

PD-1 antibodies, such as pembrolizumab (Keytruda), nivolumab (Opdivo), and cemiplimab (Libtayo), as well as PD-L1 antibodies such as atezolizumab (Tecentriq), avelumab (Bavencio), and durvalumab (Imfinzi), have been approved by the FDA to treat cancer-mediated immune exhaustion [30]. A synergistic effect of treatment with an immune checkpoint inhibitor and anti-cancer drugs was recently observed, compared with treatment with an immune checkpoint inhibitor alone; clinical trials are currently in progress [16, 31]. Many new drugs and therapeutic strategies targeting PD-1/PD-L1 are being developed, so it is important to determine the mechanism underlying the increased expression of PD-L1.

Tumors are classified as hot (immunogenic) or cold (non-immunogenic) depending on infiltrated T cells. Hot tumors with infiltrating T cells respond to immunotherapy, whereas cold tumors with little or no infiltrating T cells are resistance to immunotherapy [32]. Therefore, converting cold tumors into hot tumors can be a very important cancer treatment strategy. In a recent study, most tumors in an acidic environment were characteristic of cold tumors, but infiltered CD8+ T cells were increased when treated with alkaline reagents or pH control gel, which turned cold tumor characteristics into hot tumor characteristics [33, 34]. In addition, combination therapy of these alkaline reagents and immune checkpoint inhibitor effectively suppressed cancer. Since the progression and development of breast cancer show a positive correlation with acidosis, a good prognosis for immunotherapy can be expected if the acidic tumor microenvironment is targeted to convert the cold tumor characteristics of TNBC into hot tumor characteristics [35].

The oncogene STAT3, as well as tyrosine kinases such as members of the Janus kinase (JAK) family, are phosphorylated by cytokines or growth factors to sequentially activate STAT3 [36, 37]. In particular, the phosphorylation of tyrosine 705 of STAT3 induces expression of several target genes that exacerbate cancer progression [38, 39]. Since STAT3 is also known to induce the expression of PD-L1, it is expected to exert dual effects by inhibiting STAT3 to prevent cancer progression and targeting PD-L1 to prevent immune exhaustion. A clinical trial is currently underway on a combination therapy for metastatic colorectal cancer comprising the STAT3 inhibitor napabucasin and PD-L1 inhibitor pembrolizumab (NCT02851004) [40]. Targeting STAT3 activation will be important in cancer immunotherapy.

As STAT3, STAT1 is also well known to increase expression of PD-L1. In previous studies, it was reported that IFNs secreted by T cells activate STAT1 to increase the expression of PD-L1 in chronic hepatitis, melanoma and gastric carcinoma [41–43]. When we additionally verified the activation of STAT1 by the acidic tumor microenvironment, the expression of pY-STAT1 was not increased in acidic conditions (Supplementary Fig. 4A). Therefore, we suggested that the increase of PD-L1 by acidosis was increased by activation of STAT3.

In this study, extracellular acidosis increased PD-L1 expression (Fig. 1), as did lactic acid acidification caused by hypoxia or the Warburg effect (the primary cause of tumor acidification in cancer cells) (Fig. 2). The increased expression of PD-L1 induced by acidification was attenuated when the acidic pH was neutralized or buffered (Fig. 3). The increase in PD-L1 expression was due to acidosis-mediated STAT3 activation, and PD-L1 expression did not increase when STAT3 activation was inhibited under acidic conditions in MDA-MB-231 cells (Fig. 4). Moreover, the expression of PD-L1, pY-STAT3, HIF-1α, and LDH-A was higher in breast cancer tissue compared with normal tissue. Overall, our results suggest that extracellular acidosis increases PD-L1 expression via activation of STAT3 in MDA-MB-231 cells.

## Conclusions

In cancer cells, excessive production of lactic acid results in extracellular release, which in turn leads to extracellular acidosis. Tumor acidosis increased the expression of PD-L1 by activating STAT3 (Fig. 5D). Thus, targeting tumor acidosis may be a viable therapeutic strategy to prevent immune exhaustion, by inhibiting the increased expression of PD-L1 via STAT3.

## Supplementary Information

**Additional file 1: Supplementary Figure 1. A** The pH of the medium was adjusted by HCl or NaOH and stabilized in a 5% CO_2_ incubator. the medium pH was measured using a pH meter. **B** The pH of the medium was adjusted by HCl or NaOH, and MDA-MB-231 cells were incubated for 18 h. mRNA levels were analyzed by qPCR. **Supplementary Fig. 2. A–C** MDA-MB-231 cells were treated with various conditioned media for 18 h. Protein levels were analyzed by western blotting **(A)**, mRNA levels were analyzed by qPCR **(B)**, and the culture medium pH was measured using a pH meter **(C)***.* **D, E** MDA-MB-231 cells were treated with lactic acid, with/without, NaOH for 24 h. Protein levels were analyzed by western blotting **(D)** and the culture medium pH was measured using a pH meter **(E)**. **Supplementary Fig. 3. A** MDA-MB-231 cells were treated with lactic acid or sodium lactate, with/without, NaOH for 24 h. Protein levels were analyzed by western blotting. **B** MDA-MB-231 cells were treated with various conditioned media with/without NaOH for 18 h. Protein levels were analyzed by western blotting. **C** The summarization of the process by which various conditioned media are prepared. CON: control; fresh media, NCM: normoxic conditioned media, HCM: hypoxic conditioned media, Oxamate pre-treated HCM). **D-F** The various conditioned media were treated in MDA-MB-231 breast cancer cells for 18 h. Cells were analyzed by westernblotting **(D)** and qPCR **(E)**, and cultured media pH was measured using a pH meter **(F)**. **G** MDA-MB-231 cells were treated with a nifuroxazide under acidic conditions for 18 h. Protein levels were analyzed by western blotting. **Supplementary Fig. 4. A** Acidic media were treated in MDA-MB-231 breast cancer cells for 18 h. Cell lysates were analyzed by westernblotting. **Supplementary Fig. 5.** Densitometric and statistical analysis of all western blot data in main figures. **Supplementary Fig. 6.** Densitometric and statistical analysis of all western blot data in supplementary figures. **Supplementary Fig. 7.** Full-length blots in Figure 1. The red dotted lines are the main figures. **Supplementary Fig. 8.** Full-length blots in Figure 2. The red dotted lines are the main figures. **Supplementary Fig. 9.** Full-length blots in Figure 3. The red dotted lines are the main figures. **Supplementary Fig. 10.** Full-length blots in Figure 4. The red dotted lines are the main figures. **Supplementary Fig. 11.** Full-length blots in Supplementary Figure 2. The red dotted lines are the main figures. **Supplementary Fig. 12.** Full-length blots in Supplementary Figure 3.The red dotted lines are the main figures. **Supplementary Fig. 13.** Full-length blots in Supplementary Fig. 4.The red dotted lines are the main figures.

## Data Availability

All databases analyzed in this study were obtained from National Center for Biotechnology Information (NCBI) (https://www.ncbi.nlm.nih.gov/geo/).

## Abbreviations

ATCC: American Type Culture Collection
DMEM: Dulbecco’s modified Eagle’s medium
FBS: Fetal bovine serum
GSEA: Gene set enrichment analysis
HCM: Hypoxic conditioned medium
HIF-1α: Hypoxia-inducible factor-1α
IHC: Immunohistochemistry
JAK: Janus Kinase
LDH: Lactate dehydrogenase
LLC: Lewis lung carcinoma
MCT: Monocarboxylate transporter
NaHCO_3_: Sodium bicarbonate
NCM: Normoxic conditioned medium
PD-1: Programmed cell death protein-1
PD-L1: Programmed cell death-ligand 1
qPCR: Quantitative real-time polymerase chain reaction
STAT: Signal transducer and activator of transcription
TNBC: Triple-negative breast cancer
