# Supplementary material for: The acidic tumor microenvironment enhances PD-L1 expression via activation of STAT3 in MDA-MB-231 breast cancer cells
Source: BMC Cancer. 2022 Aug 4;22:852. doi: 10.1186/s12885-022-09956-9 (PMC9351117; doi:10.1186/s12885-022-09956-9)
Supplement: Supplementary file 1 — Additional file 1: Supplementary Figure 1. A The pH of the medium was adjusted by HCl or NaOH and stabilized in a 5% CO2 incubator. the medium pH was measured using a pH meter. B The pH of the medium was adjusted by HCl or NaOH, and MDA-MB-231 cells were incubated for 18 h. mRNA levels were analyzed by qPCR. Supplementary Fig. 2. A–C MDA-MB-231 cells were treated with various conditioned media for 18 h. Protein levels were analyzed by western blotting (A), mRNA levels were analyzed by qPCR (B), and the culture medium pH was measured using a pH meter (C). D, E MDA-MB-231 cells were treated with lactic acid, with/without, NaOH for 24 h. Protein levels were analyzed by western blotting (D) and the culture medium pH was measured using a pH meter (E). Supplementary Fig. 3. A MDA-MB-231 cells were treated with lactic acid or sodium lactate, with/without, NaOH for 24 h. Protein levels were analyzed by western blotting. B MDA-MB-231 cells were treated with various conditioned media with/without NaOH for 18 h. Protein levels were analyzed by western blotting. C The summarization of the process by which various conditioned media are prepared. CON: control; fresh media, NCM: normoxic conditioned media, HCM: hypoxic conditioned media, Oxamate pre-treated HCM). D-F The various conditioned media were treated in MDA-MB-231 breast cancer cells for 18 h. Cells were analyzed by westernblotting (D) and qPCR (E), and cultured media pH was measured using a pH meter (F). G MDA-MB-231 cells were treated with a nifuroxazide under acidic conditions for 18 h. Protein levels were analyzed by western blotting. Supplementary Fig. 4. A Acidic media were treated in MDA-MB-231 breast cancer cells for 18 h. Cell lysates were analyzed by westernblotting. Supplementary Fig. 5. Densitometric and statistical analysis of all western blot data in main figures. Supplementary Fig. 6. Densitometric and statistical analysis of all western blot data in supplementary figures. Supplementary Fig. 7. Full-length [file 12885_2022_9956_MOESM1_ESM.docx]

*Supplementary information*

The acidic tumor microenvironment enhances PD-L1 expression via activation of STAT3 in MDA-MB-231 breast cancer cells

**Yong-Jin Kwon, et al.**


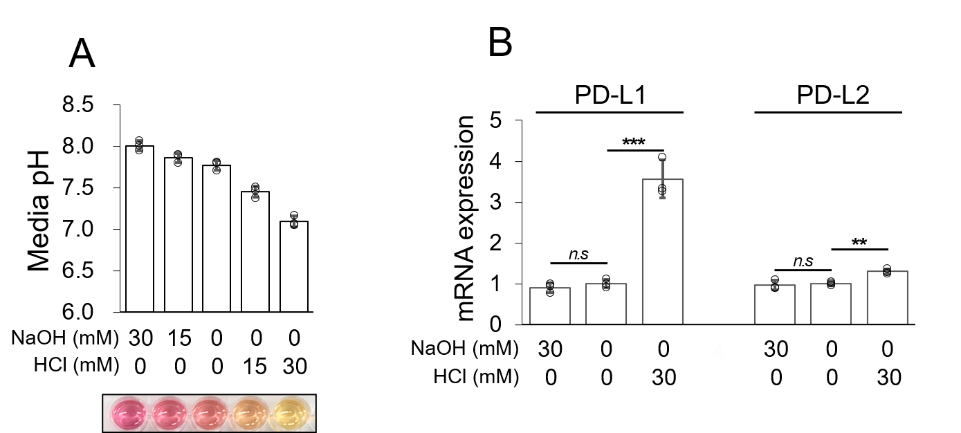


**Supplementary Figure 1.**

**A** The pH of the medium was adjusted by HCl or NaOH and stabilized in a 5% CO_2_ incubator. the medium pH was measured using a pH meter. **B** The pH of the medium was adjusted by HCl or NaOH, and MDA-MB-231 cells were incubated for 18 h. mRNA levels were analyzed by qPCR.


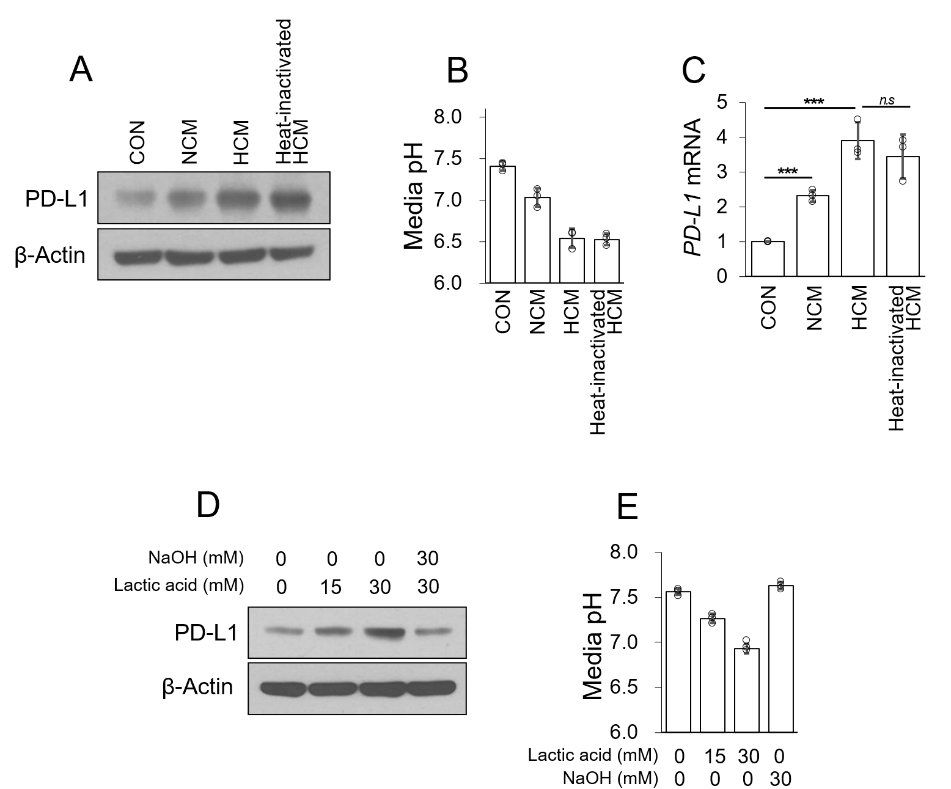


**Supplementary Fig. 2**

**A–C** MDA-MB-231 cells were treated with various conditioned media for 18 h. Protein levels were analyzed by western blotting **(A)**, mRNA levels were analyzed by qPCR **(B)**, and the culture medium pH was measured using a pH meter **(C)***.* **D, E** MDA-MB-231 cells were treated with lactic acid, with/without, NaOH for 24 h. Protein levels were analyzed by western blotting **(D)** and the culture medium pH was measured using a pH meter **(E)**.


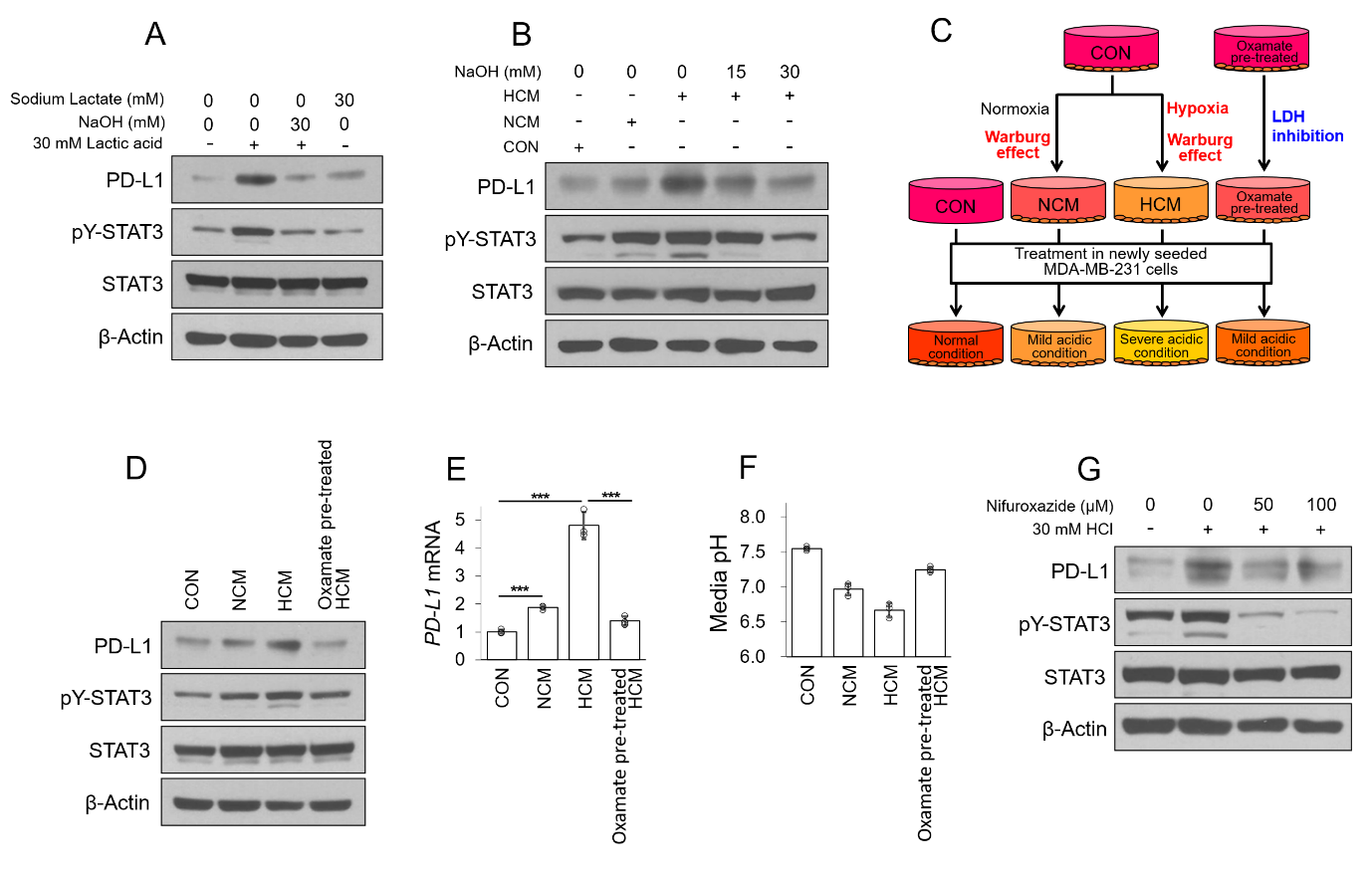


**Supplementary Fig. 3.**

**A** MDA-MB-231 cells were treated with lactic acid or sodium lactate, with/without, NaOH for 24 h. Protein levels were analyzed by western blotting. **B** MDA-MB-231 cells were treated with various conditioned media with/without NaOH for 18 h. Protein levels were analyzed by western blotting. **C** The summarization of the process by which various conditioned media are prepared. CON: control; fresh media, NCM: normoxic conditioned media, HCM: hypoxic conditioned media, Oxamate pre-treated HCM). **D-F** The various conditioned media were treated in MDA-MB-231 breast cancer cells for 18 h. Cells were analyzed by westernblotting **(D)** and qPCR **(E)**, and cultured media pH was measured using a pH meter **(F)**. **G** MDA-MB-231 cells were treated with a nifuroxazide under acidic conditions for 18 h. Protein levels were analyzed by western blotting.


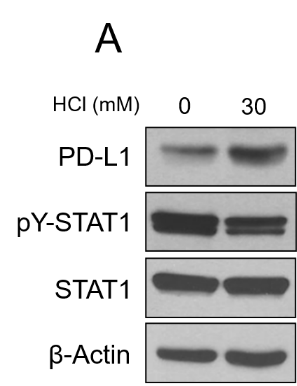


**Supplementary Fig. 4.**

**A** Acidic media were treated in MDA-MB-231 breast cancer cells for 18 h. Cell lysates were analyzed by westernblotting


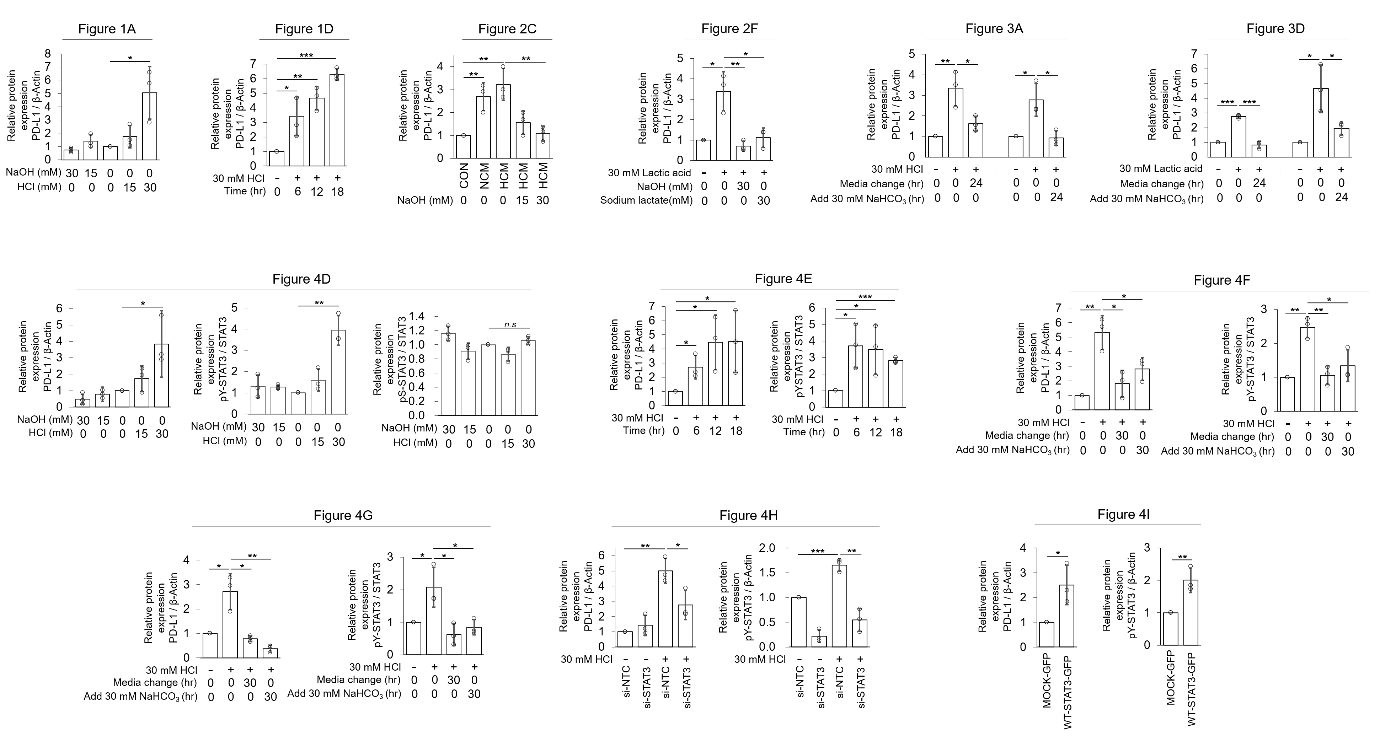


**Supplementary Fig. 5.**

Densitometric and statistical analysis of all western blot data in main figures


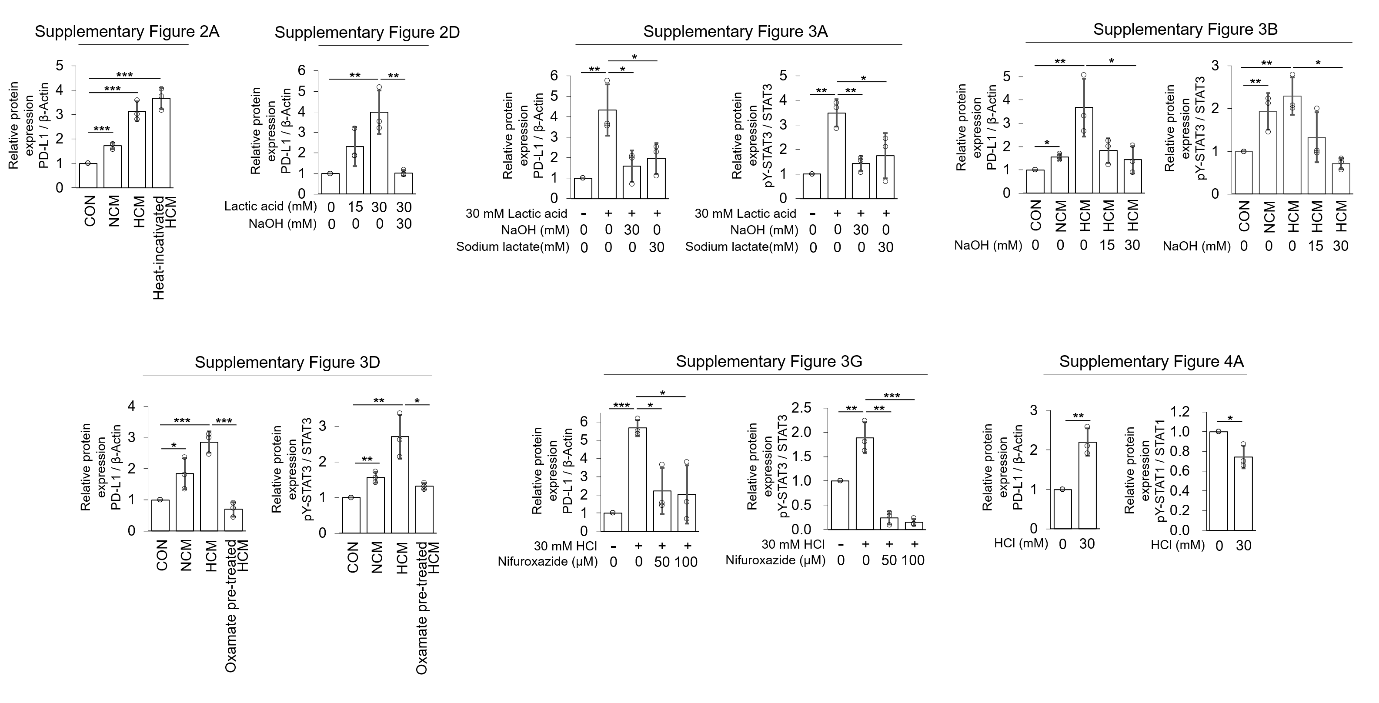


**Supplementary Fig. 6.**

Densitometric and statistical analysis of all western blot data in supplementary figures


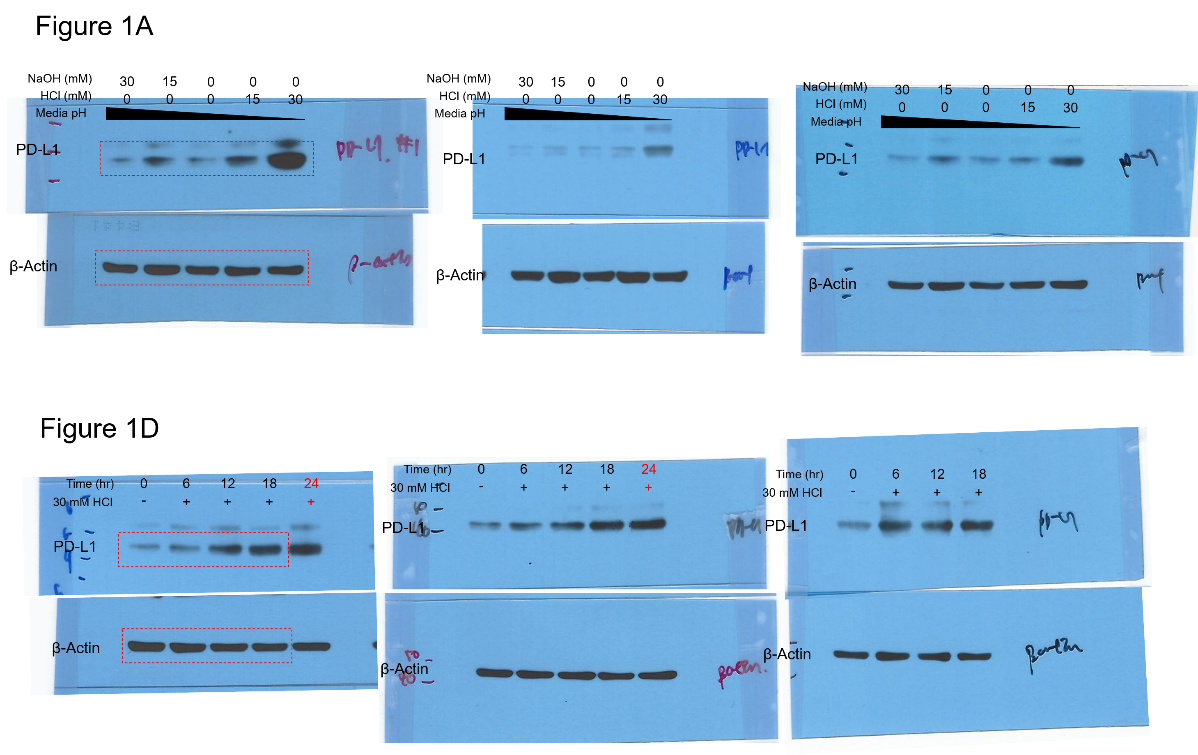


**Supplementary Fig. 7.**

Full-length blots in Figure 1. The red dotted lines are the main figures.


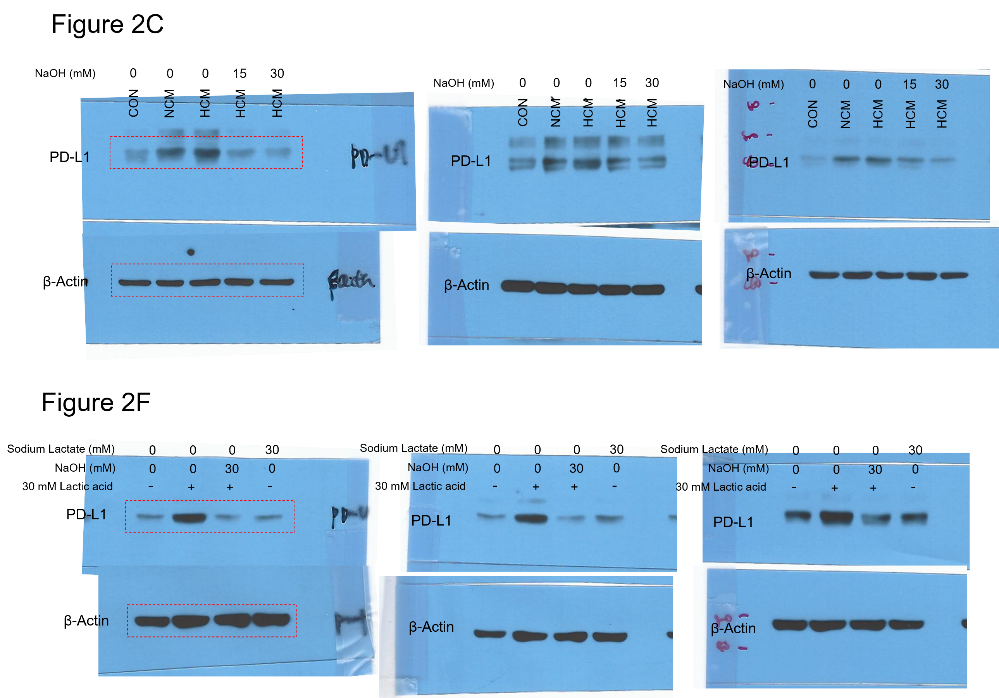


**Supplementary Fig. 8.**

Full-length blots in Figure 2. The red dotted lines are the main figures.


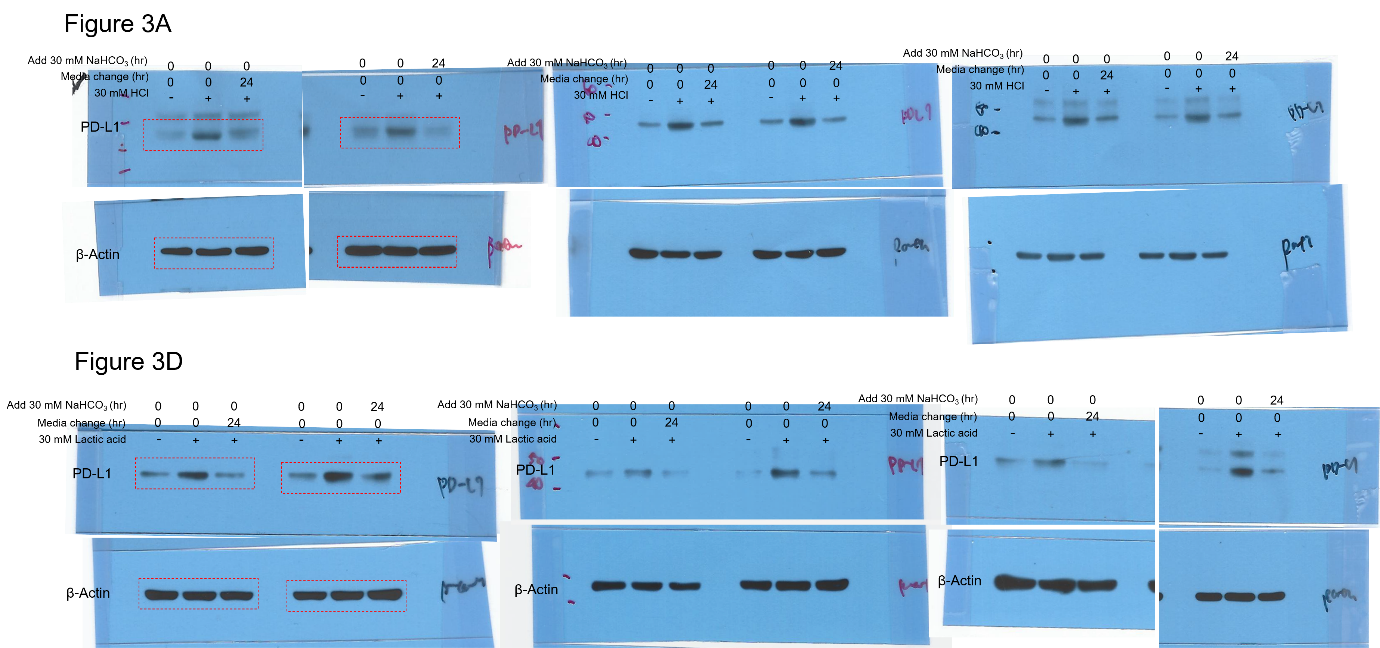
 **Supplementary Fig. 9.**

Full-length blots in Figure 3. The red dotted lines are the main figures.


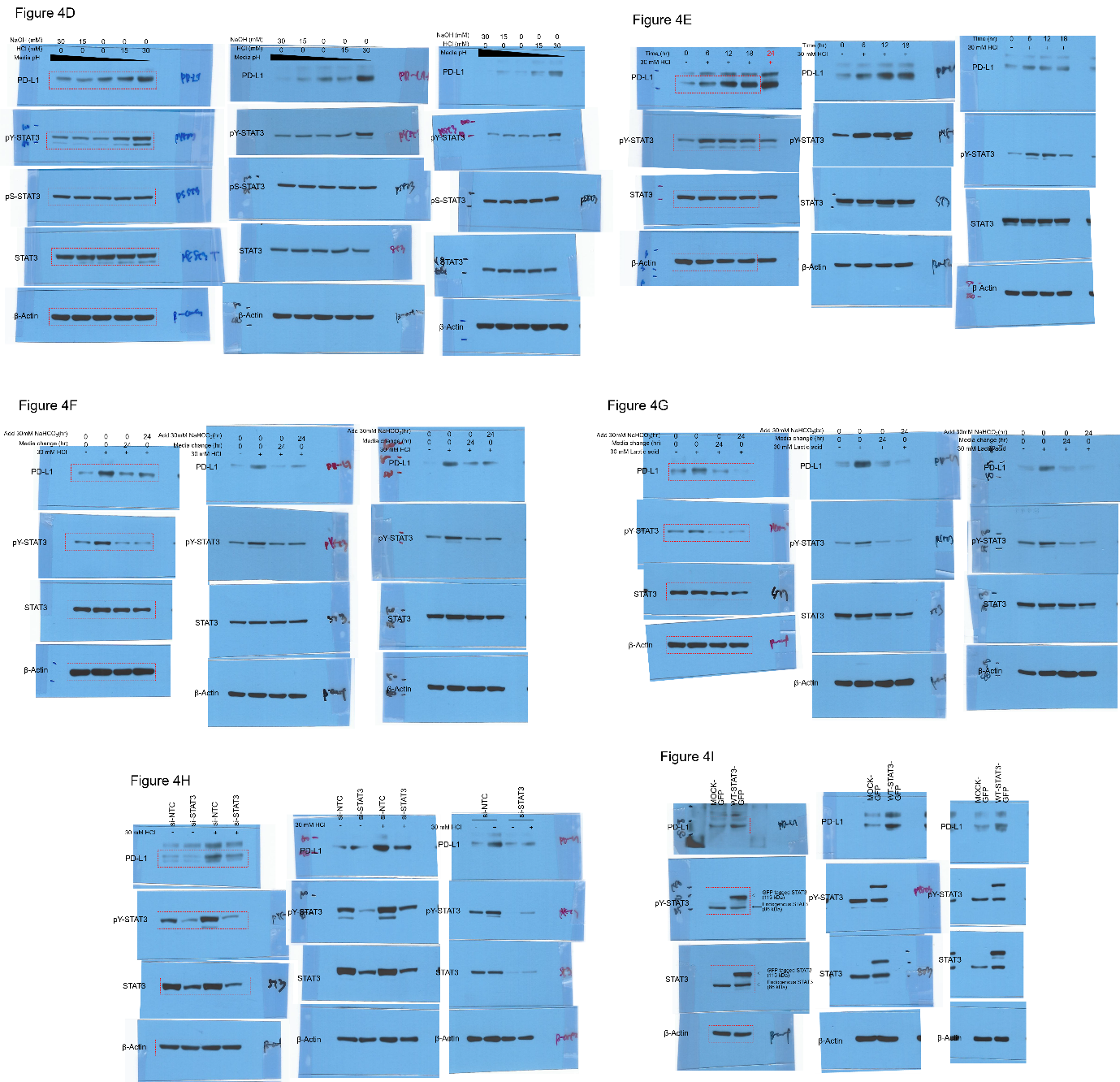


**Supplementary Fig. 10.**

Full-length blots in Figure 4. The red dotted lines are the main figures.


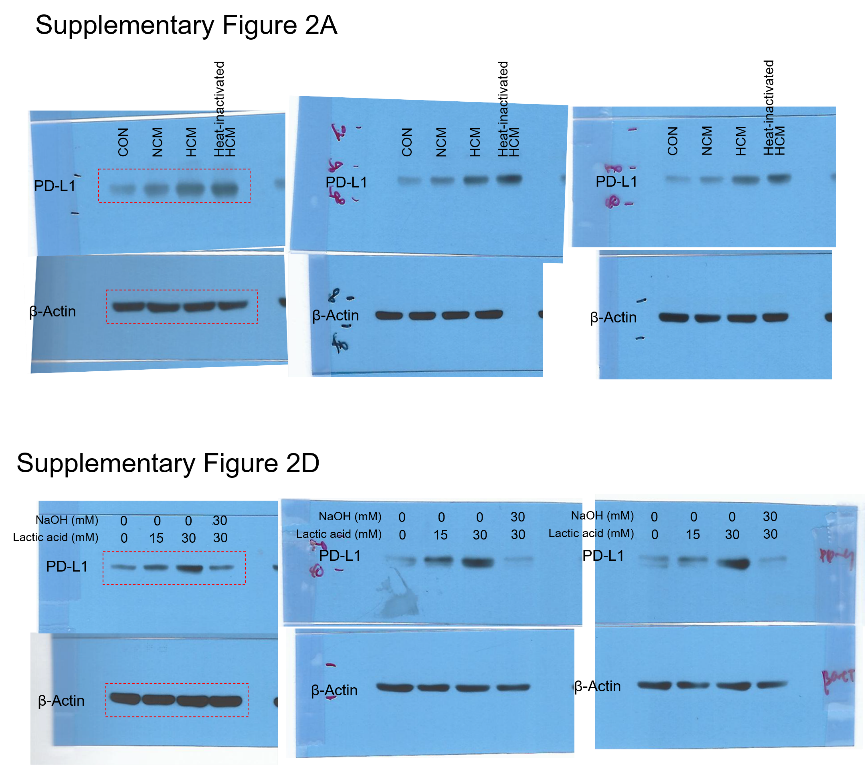


**Supplementary Fig. 11.**

Full-length blots in Supplementary Figure 2. The red dotted lines are the main figures.


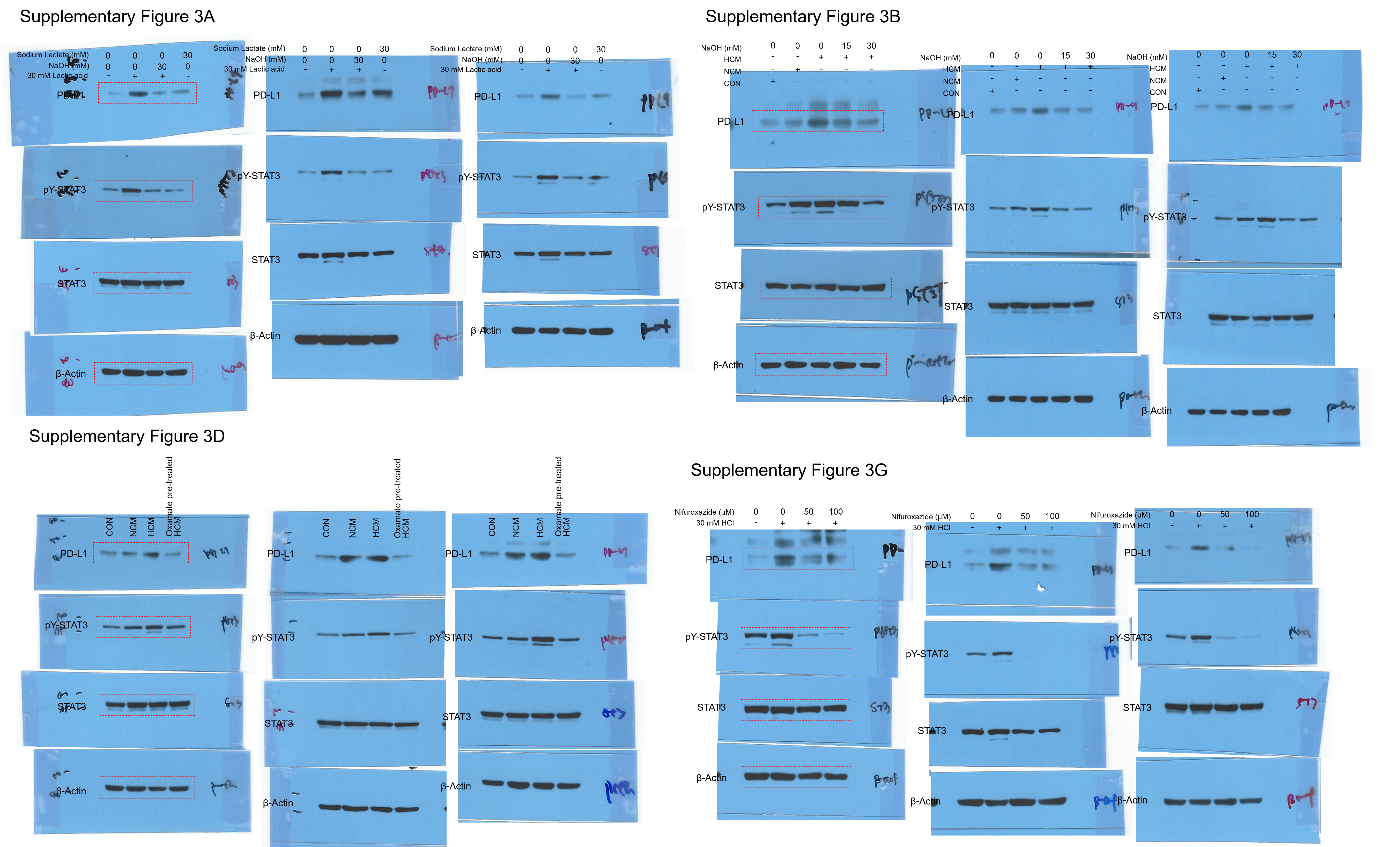


**Supplementary Fig. 12.**

Full-length blots in Supplementary Figure 3.The red dotted lines are the main figures.


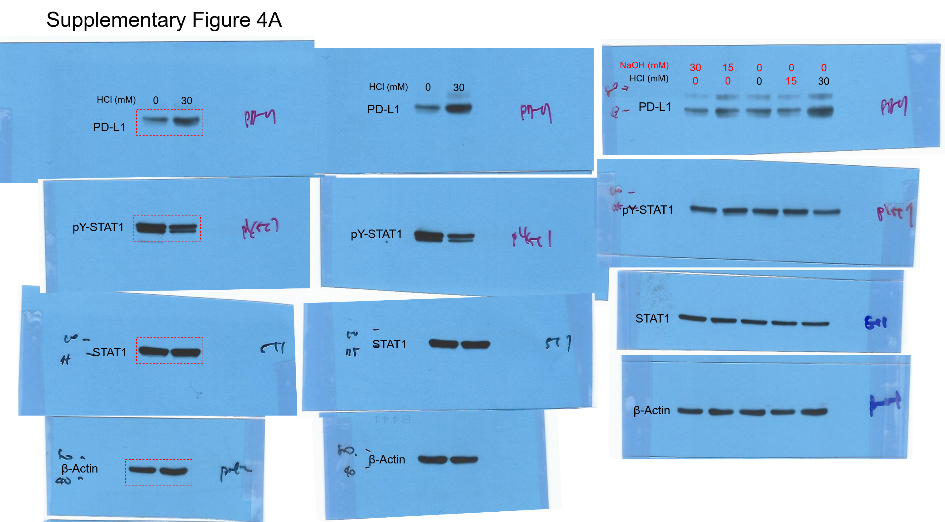


**Supplementary Fig. 13.**

Full-length blots in Supplementary Figure 4.The red dotted lines are the main figures.
